# Left-truncated effects and overestimated meta-analytic means

**DOI:** 10.1073/pnas.2203616119

**Published:** 2022-07-19

**Authors:** Jonathan Z. Bakdash, Laura R. Marusich

**Affiliations:** ^a^US Army Combat Capabilities Development Command Army Research Laboratory South, University of Texas at Dallas, Richardson, TX 75080;; ^b^Department of Psychology and Special Education, Texas A&M–Commerce, Commerce, TX 75428;; ^c^US Army Combat Capabilities Development Command Army Research Laboratory South, University of Texas at Arlington, Arlington, TX 76019

The meta-analytic by Mertens et al. (1) interprets nudges as a generally effective technique for increasing desirable decision-making, with an overall pooled effect size of *d* = 0.43. This research also reports large systematic variations (meta-analytic heterogeneity) in effects, primarily attributed to moderators such as the domain, as well as asymmetrically distributed effects, interpreted as moderate publication bias.

Apart from publication bias, non-normality and high heterogeneity may be problematic for the representativeness of meta-analytic means (2). Here, we reanalyze the corrected data made available by Mertens et al. (1), finding evidence that nudges have more limited than general effectiveness. We show that effects are clearly left-truncated, likely due to substantial publication bias, consistent with another reanalysis (3). We also find that most of the pooled effects as reported in Mertens et al. (1) are overestimated and hence unrepresentative.

First, we visualize the distributions of effects, by domain, using raincloud plots (4); see Fig. 1. Four domains (finance, food, other, and prosocial) show a concerning pattern of sharply left-truncated tails at or slightly below zero. The two remaining domains only have a handful of effects slightly below zero. A plausible mechanism for this left “cliff” is suppression of unfavorable results (5). Most domains also exhibit long right tails—a limited number of effects with large and very large magnitudes. This pattern of left truncation and long right tails strongly indicates that publication bias is greater than moderate.

**Fig. 1.**
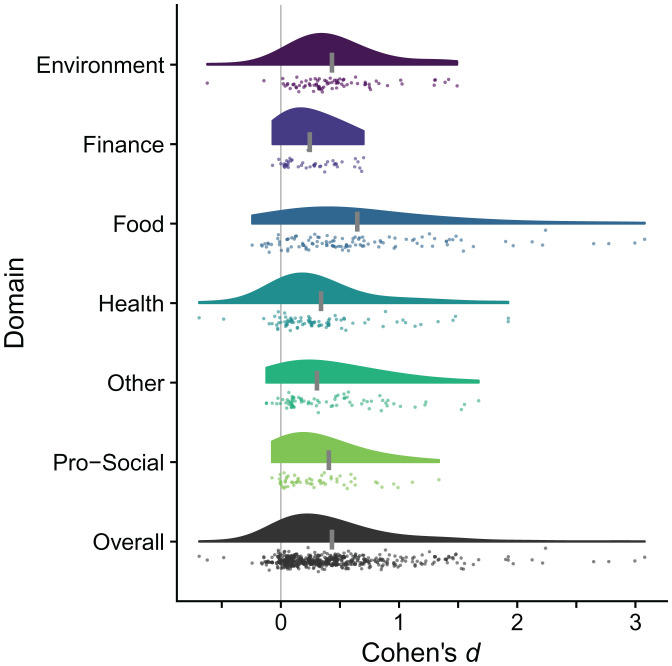
Raincloud plots of individual effects by domain and all effects. The rain is the reported effects from papers, jittered vertically, and the cloud is the smoothed distribution of effects. The short, wide, vertical gray lines on each cloud depict the corresponding meta-analytic mean. The single tall thin vertical gray line is an effect size of zero.

Second, we evaluate non-normality and the representativeness of pooled effects by domain (Table 1). Normality was assessed using Egger’s regression test for asymmetry (6). Representativeness was tested by quantifying the estimated proportion of effects below meaningful thresholds (7), here, the meta-analytic means. A perfectly representative (meta-analytic) mean would have 50% of values below it.

**Table 1.** Normality of effects and representativeness of meta-analytic effects

** **	Egger’s	Meta-analytic	Proportion of
	regression	mean	effects
Domain	test (*P* value)	(Cohen’s *d*)	below (%)
Environment	<0.001	0.43	55.26
Finance	0.01	0.24	55.56
Food	0.01	0.65	60.36
Health	<0.001	0.34	72.62
Other	<0.001	0.31	49.32
Prosocial	<0.001	0.41	67.39*
Overall	<0.001	0.43	62.64

^*^For prosocial, the proportion of effects below is underestimated because 12 effects with a Cohen’s |*d*| < 0.04 out of 58 effects were removed due to estimation problems.

All domains exhibited asymmetry, and all but one (other) had some overestimation in pooled effects, that is, a greater than expected proportion of effects below their meta-analytic mean. Despite left truncation of effects, nearly two-thirds of all effects were still below the overall meta-analytic mean.

Funnel plots can often be difficult to interpret (8), and, typically, all effects are plotted together; thus, the severity and nature of the non-normality in effects, especially by domain, may not be apparent in Mertens et al. (1). Here, we evaluate effects by domain; therefore, our results cannot be solely attributed to the heterogeneity and non-normality potentially caused by combining domains.

The end goal of nudges and related behavioral interventions is increasing desirable decision-making. Achieving this requires identifying factors associated with positive impacts, but also factors that have minimal and even negative effects on decisions (9, 10). Publication bias impedes understanding for variations in nudge effectiveness.

## Data Availability

Data and code are available at https://osf.io/jydb7/ (11) and https://codeocean.com/capsule/3133766/tree/v1 (12).
